# Etiology and Outcome of non-immune Hydrops Fetalis in Southern China: report of 1004 cases

**DOI:** 10.1038/s41598-019-47050-6

**Published:** 2019-07-24

**Authors:** Dahua Meng, Qifei Li, Xuehua Hu, Lifang Wang, Shuyin Tan, Jiasun Su, Yue Zhang, Weijia Sun, Biyan Chen, Sheng He, Fei Lin, Bobo Xie, Shaoke Chen, Pankaj B. Agrawal, Shiyu Luo, Chunyun Fu

**Affiliations:** 1 Department of Clinical Genetics, Maternal and Child Health Hospital of Guangxi Zhuang Autonomous Region, Nanning, 530003 China; 2 Division of Newborn Medicine, Boston Children’s Hospital and Harvard Medical School, Boston, 02115 USA; 3 Division of Genetics and Genomics, Boston Children’s Hospital and Harvard Medical School, Boston, MA 02115 USA; 4 The Manton Center for Orphan Disease Research, Boston Children’s Hospital and Harvard Medical School, Boston, MA 02115 USA; 5 Medical Science Laboratory, Maternal and Child Health Hospital of Guangxi Zhuang Autonomous Region, Nanning, 530003 China; 6 Department of Genetic Metabolism, Maternal and Child Health Hospital of Guangxi Zhuang Autonomous Region, Nanning, 530003 China; 7 Research Center for Guangxi Birth Defects Control and Prevention, Maternal and Child Health Hospital of Guangxi Zhuang Autonomous Region, Nanning, 530003 China

**Keywords:** Endocrine system and metabolic diseases, Epidemiology

## Abstract

Non-immune hydrops fetalis (NIHF) is a complex condition with a high mortality and morbidity rate. Here we report the etiology and outcome of 1004 fetuses with NIHF, in a large single Maternal and Children’s hospital of Southern China, since the year of 2009 to 2016. Among these 1004 fetuses with NIHF, the etiology was identified prenatally in 722 of them (72%). The most common ones were hematologic diseases and chromosomal abnormalities. There were eight spontaneous abortions, 18 intrauterine fetal demise, 672 pregnancy terminations and 87 were lost to follow-up. 219 of the 1004 fetuses were live-born and the overall survival rate was 21.8% at this point. After birth 16 perinatal or early neonatal deaths were encountered and five lost to follow-up. Of the remaining 198 newborns, 153 thrived without apparent morbidity. The most significant factors associated with mortality were prematurity and low birthweight. In conclusion, we described the largest report of underlying causes and outcome of NIHF in Southern China. Etiologies were identified for 72% of 1004 fetuses with NIHF. And two poor prognostic factors for survival are preterm birth at less than 36.5 weeks and birthweight lower than 2575 g respectively.

## Introduction

Hydrops fetalis (HF) refers to a condition in which an excess of fluid accumulates in fetal soft tissues and serous cavities^1–3^. According to the clinical guideline from the Society for Maternal-Fetal Medicine^4^, HF is defined as the presence of at least two abnormal fluid collections in the fetus, including ascites, pericardial effusion, pleural effusions, and generalized skin edema (skin thickness >5 mm). Polyhydramnios and placental thickening are also frequent sonographic findings^5,6^. Generally, HF could be classified into two categories: iso-immune HF (IHF) and non-immune HF (NIHF). IHF is caused by fetal hemolysis, an process that is mediated by circulating maternal antibodies to antigens of fetal red blood cells. The incidence of IHF has markedly declined due to the use of anti-D gamma globulin prophylaxis. At present, about 90% of cases of hydrops are NIHF^7^, with prevalence reported as 1 in 1700–3000 pregnancies^8–10^.

NIHF may occur at various gestational ages with different types of etiologies, including hematologic, cardiovascular, chromosomal, infections, syndromic, thoracic, inborn errors of metabolism, lymphatic dysplasia, urinary tract malformations, extra thoracic tumors, gastrointestinal, twin-to-twin transfusion syndrome (TTTS)-placental, miscellaneous, and idiopathic causes^1,11^. Despite many improvements in diagnosis and management, however, the mortality rate of NIHF remains high during either fetal or neonatal period, and predicting survival still poses a challenge^12–17^. The prognosis for NIHF varies based on the underlying etiologies and gestational ages at diagnosis and at delivery^18,19^, thus clarifying the primary etiology is crucial for genetic counseling and future pregnancy management. Up to date, there have been limited studies into the prevalence and etiology of NIHF in China^20^, and the association between the etiology and either perinatal or developmental outcome has not been fully determined. The aim of this study was to describe the etiologies and outcome of 1004 NIHF cases in Southern China, and evaluate the relationship among the etiology, clinical characteristics, and outcome.

## Results

### Demographic data

In this study, 1004 cases of NIHF were diagnosed in a total of 126,856 pregnancies (incidence of 7.9/1000) by antenatal ultrasound scanning. The median maternal age was 28 years (IQR 25–32 years), and the median gestational age (GA) at diagnosis of NIHF was 23 weeks (IQR 17–26 weeks).

### Etiology

Table 1 lists the etiologies of these 1004 fetuses with NIHF. The causes of NIHF in these cases were identified prenatally in 722 of them (72.0%), but could not be ascertained in the remaining 282 cases (28.0%). The most common causes of NIHF in our study were hematologic diseases (*n* = 285, 28.4%) and chromosomal abnormalities (*n* = 199, 19.8%), followed by lymphatic anomalies (*n* = 78, 7.8%), cardiovascular disorders (*n* = 41, 4.1%), twin-to-twin transfusion syndrome (TTTS) – placental/cord problems (*n* = 30, 3.0%), urinary tract malformations (*n* = 29, 2.9%), intrauterine infections (*n* = 26, 2.6%), thoracic malformations (*n* = 17, 1.7%), miscellaneous causes (*n* = 8, 0.8%), gastrointestinal disorders (*n* = 7, 0.7%) and syndromic diseases (*n* = 2, 0.2%).

**Table 1:** Etiology of 1004 cases of non-immune hydrops fetalis.

Classifications	Number of cases	Pregnancy outcome								Neonatal outcome					Overall survival rate (%)
		natural labour	cesarean section	premature labour	spontaneous abortion	induced abortion	fetal demise	lost to follow-up	Prenatal survival rate (%)	intact survivors	co-morbidity survivors	PND or END	lost to follow-up	Intact survival rate (%)	
Hematologic disease	**285**	3	2	1	0	269	0	10	**2**.**2**	2	1	2	1	**40**.**0**	**1**.**1**
Chromosomal abnormalities	**199**	1	0	0	0	189	4	5	**0**.**5**	0	1	0	0	**0**.**0**	**0**.**5**
Lymphatic anomalies	**78**	14	14	0	1	36	0	13	**43**.**1**	11	16	0	1	**40**.**7**	**42**.**2**
Cardiovascular disorders	**41**	4	5	1	0	23	1	7	**29**.**4**	5	3	2	0	**50**.**0**	**23**.**5**
TTTS-placental/cord problems	**30**	1	4	1	3	10	8	3	**22**.**2**	4	1	1	0	**66**.**7**	**18**.**5**
Urinary tract malformations	**29**	9	5	0	0	12	0	3	**53**.**8**	6	5	3	0	**42**.**9**	**42**.**3**
Intrauterine infections	**26**	7	1	1	0	14	0	3	**39**.**1**	7	1	1	0	**77**.**8**	**34**.**8**
Thoracic malformations	**17**	2	0	0	0	13	1	1	**12**.**5**	1	1	0	0	**50**.**0**	**12**.**5**
Miscellaneous	**8**	2	1	0	0	5	0	0	**37**.**5**	3	0	0	0	**100**.**0**	**37**.**5**
Gastrointestinal disorders	**7**	3	0	2	1	1	0	0	**71**.**4**	5	0	0	0	**100**.**0**	**71**.**4**
Syndromic diseases	**2**	0	0	1	0	1	0	0	**50**.**0**	0	0	1	0	**0**.**0**	**0**.**0**
Idiopathic causes	**282**	80	47	7	3	99	4	42	**55**.**8**	109	16	6	3	**83**.**2**	**52**.**7**
Total	**1004**	**126**	**79**	**14**	**8**	**672**	**18**	**87**	**23**.**9**	**153**	**45**	**16**	**5**	**71**.**5**	**21**.**7**

In 285 cases of hematologic diseases, 267 cases were caused by typical Bart’s hemoglobin disease, 17 were caused by hemoglobin H disease associated with α-globin gene Quong Sze mutation, Constant Spring mutation and Westmead variant, and one case by α compound β thalassemia gene mutations. Among 199 cases of chromosomal abnormalities, there were 183 cases of chromosomal number abnormalities, 5 cases of chromosomal structure abnormalities and 11 cases of copy number variations, involving mostly chromosomes X, 21, 18 and 13. Among the group of intrauterine infections, there were 11 cases with *Cytomegalovirus*, 9 cases with *Hepatitis B* virus, 3 cases with *Parvovirus* B19, 2 cases with *Herpes simplex* virus and 1 case with *Syphilis*. See Appendix Table 1 for more details.

### Etiology and clinical characteristics

**Figure 1:**
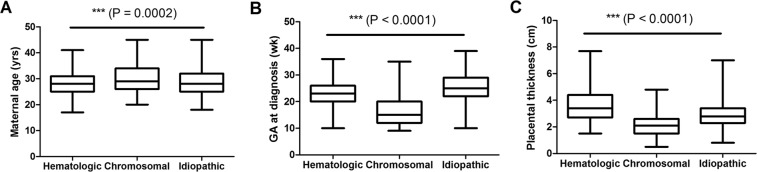
Comparison of clinical parameters among the groups of hematologic, chromosomal and idiopathic diseases. (**A**) Maternal age (yrs), (**B**) GA at diagnosis (wk), (**C**) Placental thickness (cm).

The clinical parameters were significantly different among three groups (hematologic, chromosomal and idiopathic) with appropriate sampling size, including maternal age (*P* = 0.0002), GA at diagnosis (*P* < 0.0001) and placental thickness (*P* < 0.0001) (Fig. 1). The median maternal age of these three groups were 28 years (IQR 25–31 years), 29 years (IQR 26–34 years) and 28 years (IQR 25–32 years), respectively. The GAs at the diagnosis of NIHF were 23 weeks (IQR 20–26 weeks), 15 weeks (IQR 12–20 weeks) and 25 weeks (IQR 22–29 weeks). The placental thickness were 3.6 ± 1.2 cm, 2.1 ± 0.8 cm and 3.0 ± 0.9 cm. Appendix Table 2 lists top 10 ultrasound findings among 1004 NIHF cases with different etiologic classifications. Hematologic diseases were often associated with increased cardiothoracic ratio (206/285, 72.3%), ascites (116/285, 40.7%), intestinal echo enhancement and pericardial effusion (113/285, 39.6%), whereas chromosomal abnormalities were associated with skin edema (65.8%) and cystic hygroma (49.2%).

### Outcome

**Figure 2:**
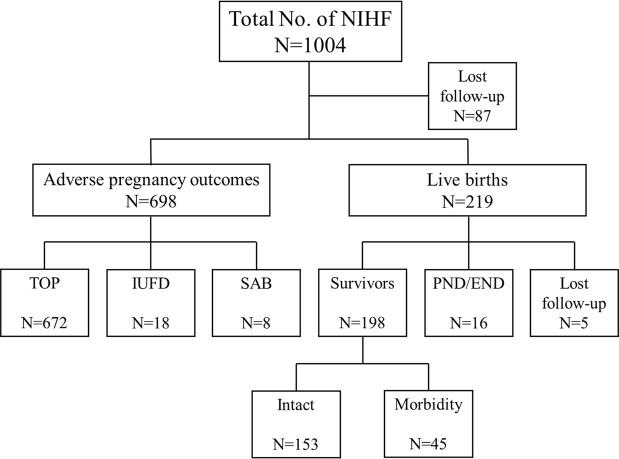
The patient pathway in the current study.

There were eight spontaneous abortions, 18 intrauterine fetal demise (IUFD) and 672 pregnancy terminations. Eighty seven pregnancies were lost to follow-up. A total of 219 of the 1004 fetuses (21.8%) survived till birth with a median GA at delivery of 38 weeks (IQR 37–40 weeks) and a mean birth weight of 2938 ± 554 g. Six percent of infants (14 of 219) were premature and thirty-six percent (79 of 219) were delivered via cesarean section. Five cases were lost to follow-up after birth. In the remaining 214 cases with follow-up, there were 16 perinatal deaths (PND) or early neonatal deaths (END). Overall, a total of 198 (198/1004, 19.7%) infants survived without mortality (Fig. 2), of which 153 intact survivors with no significant morbidity had NIHF mostly because of idiopathic causes (*n* = 109), lymphatic anomalies (*n* = 11), intrauterine infections (*n* = 7) or urinary tract malformations (*n* = 6) (Table 1). In addition, 45 cases had some co-morbidity that differed from some mild conditions, such as cystic hygroma or intestinal obstruction that could be treated by surgical therapy, to severe neurodevelopmental delay. The mean duration of follow-up was about one year after delivery.

### Prognostic factors

**Figure 3:**
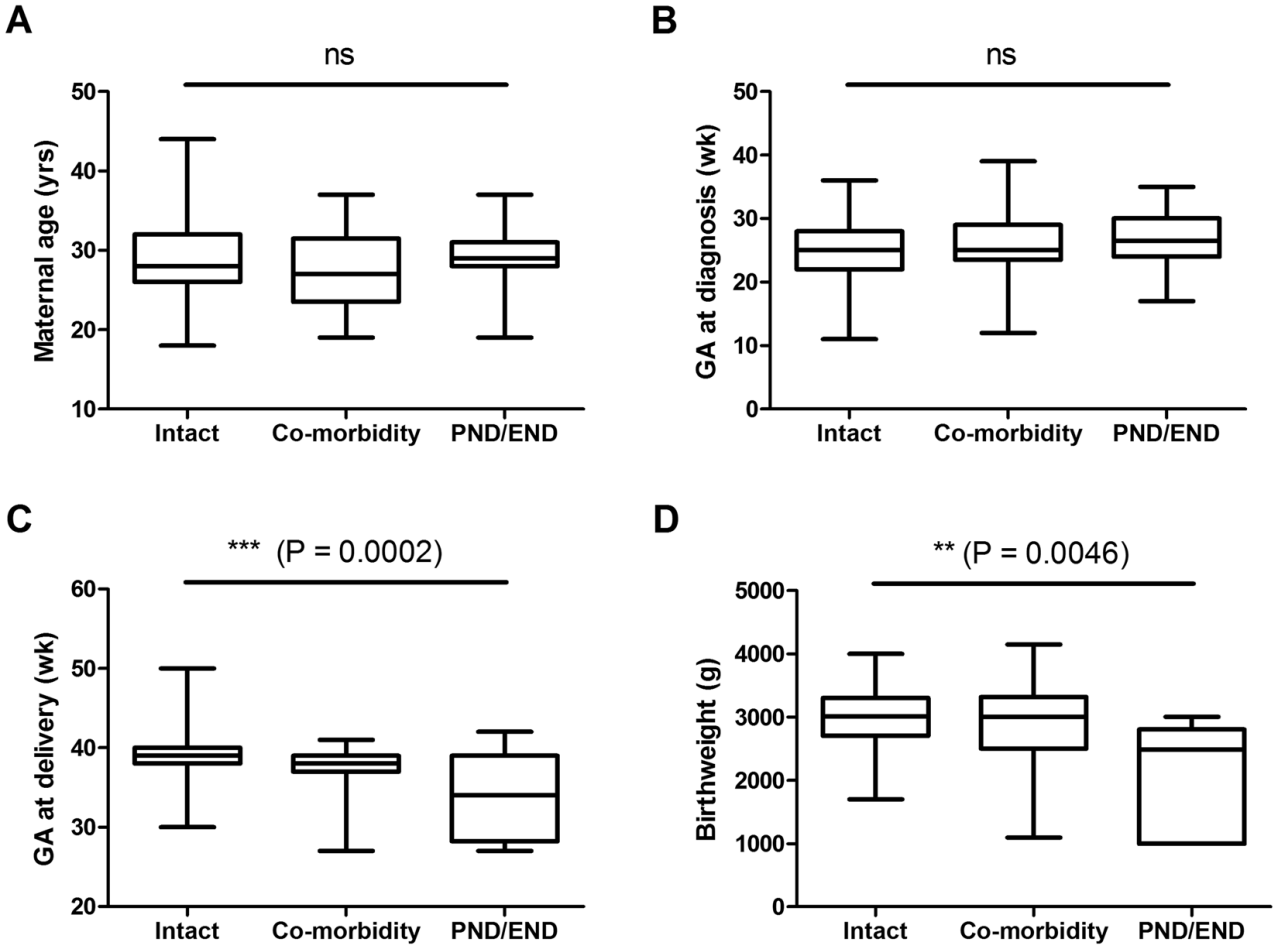
Comparison of clinical parameters among the groups of intact survivors, survivors with some co-morbidity and cases of fetal demise. (**A**) Maternal age (yrs), (**B**) GA at diagnosis (wk), (**C**) GA at delivery (wk), (**D**) Birthweight (g). PND: perinatal deaths, END: early neonatal deaths.

The overall survival rate was highest among neonates with gastrointestinal (71.4%, *n* = 7), idiopathic (52.7%, *n* = 282), urinary tract (42.3%, *n* = 29) and lymphatic defects (42.2%, *n* = 78) causes, and lowest among neonates with syndromic (0.2%, *n* = 2), chromosomal (0.5%, *n* = 199) and hematologic (1.1%, *n* = 285) diseases (Table 1). By univariate analysis (as shown in Fig. 3 and Table 2), the risk factors for mortality are early GAs at delivery (*P* = 0.0002) and lower birthweight (*P* = 0.0046). The median GAs at delivery in the group of intact survivors, co-morbidity survivors, and fatal cases were 39 weeks (IQR 38–40 weeks), 38 weeks (IQR 37–39 weeks) and 34 weeks (IQR 28–39 weeks) and the mean birthweight were 2998 ± 468 g, 2878 ± 642 g and 1997 ± 903 g respectively. By using ROC curve analysis, the area under the curve is 0.76 for GAs at delivery and 0.86 for birthweight, respectively. The cutoff values of GA at delivery and birthweight are 36.5 weeks (sensitivity 68.75%, specificity 85.43%) and 2575 g (sensitivity 71.43%, specificity 85.14%), respectively.

**Table 2:** Comparison of clinical characteristics among intact survivors, co-morbidity survivors and fatal cases with NIHF.

Characteristic	Intact survivors (*n* = 153)	Co-morbidity survivors (*n* = 45)	PND/END (*n* = 16)	*P*
Maternal age (yrs)	28.9 ± 5.2	27.7 ± 4.5	29.3 ± 3.7	0.2599
**Gestational age (wk)**				
At diagnosis	24.8 ± 5.5	26.0 ± 5.9	26.4 ± 4.8	0.3551
At birth	38.5 ± 2.5	37.3 ± 2.9	33.8 ± 5.1	**0.0002**
Gestational age ≤36 wks	22 (14.4%)	10 (22.2%)	10 (62.5%)	
**Delivery mode**				0.1182
Natural	103 (67.3%)	24 (53.3%)	10 (62.5%)	
Cesarean section	50 (32.7%)	21 (46.7%)	6 (37.5%)	
**Gender**				0.0731
Male	72	28	8	
Female	77	16	6	
Unknown	4	1	2	
Birthweight (g)	2998 ± 468	2878 ± 642	1997 ± 903	**0.0046**

## Discussion

Using a long-term single center dataset, we have presented the largest report of underlying causes and outcome of NIHF in Southern China. The incidence of NIHF has been reported to be 1 in 1700–3000 pregnancies in Western countries. However, limited epidemiologic investigations about the occurrence of NIHF in China have been reported. In this study, 1004 cases with NIHF were diagnosed among 126,856 pregnancies encountered at a single Maternal and Children’s hospital from a southern region of China since the year of 2009 to 2016. This high prenatal incidence (7.9/1000) of fetal NIHF could be well explained by the differences of regions and GAs at diagnosis^20^.

In this study, the causes of NIHF could be identified in 72.0% of cases prenatally. The most common diagnoses associated with NIHF were hematologic disease (28.4%) and chromosomal abnormalities (19.8%) followed by unexplained NIHF (28.0%), lymphatic (7.8%), cardiovascular (4.1%), TTTS – placental/cord problems (3.0%), urinary tract malformations (2.9%), intrauterine infections (2.6%), thoracic malformations (1.7%), miscellaneous causes (0.8%), gastrointestinal disorders (0.7%) and syndromic diseases (0.2%). The high proportion of fetuses with hematologic disease and chromosomal abnormalities were caused by the regional difference and inclusion of pregnancies in the first trimester, as is the case with high incidence of NIHF in this area. We didn’t identify any cases with inborn errors of metabolism or extra thoracic tumors due to certain limitations of our diagnostic workflow. The maternal age and GA at the diagnosis of NIHF in the group of chromosomal abnormalities is very different from other groups, as expected^21^.

Although early diagnoses have been achieved in many cases allowing for the option of antenatal therapy, the overall survival rate was 21.8% and the prognosis depended on the cause, similar to previous reports^19,22,23^. The mortality rate was highest among neonates with syndromic (100%), chromosomal (99.5%) and hematologic (98.9%) diseases. In two cases with syndromic diseases, one with thanatophoric dysplasia were selectively terminated by the parents and one with acardiac twins sequence was delivered through premature labor and ended with perinatal death. The high mortality rate in the group of chromosomal and hematologic diseases were much in accordance with a high prenatal mortality rate (including spontaneous abortions, induced abortions and fetal demise). In our study, the parents opted to terminate the pregnancy without a guaranteed good prognosis, especially when a related genetic cause has been identified^20^. The intact survival rate was 71.5%, slightly higher than other reports^12,14,15,17^. This may be due to a lack of formal assessment of neurodevelopmental outcome after patient discharge from the hospital. Instead, information from parents and records of physical evaluation at birth describing the status of the baby as healthy was relied upon. Thus, it is less likely that severe developmental delay would be classified as healthy.

Various prognostic indicators at antenatal and postnatal stages have been described in studies of NIHF, including prenatal diagnosis of NIHF, GA at delivery, Apgar scores at 1 and 5 minutes, intubation and chest compression in the delivery room, the presence of pleural effusion, ≥2 cavity effusions, low serum albumin concentration, and need for thoracentesis^13–15,17–19,21,24^. Among all these factors, GA at delivery has been identified as a prognostic factor in several findings^13,18,19,21^. In our study, preterm GA at delivery and lower birthweight were remarkably associated with the risk of postnatal deaths, whereas other factors including maternal age, GA at diagnosis, delivery mode and gender, were non-significant.

In conclusion, we retrospectively summarized the etiology and outcome of 1004 cases with NIHF in Southern China. Although the prevalence and etiologic causes vary greatly among different country regions and different studied populations, our study could thereby provide much specific information for genetic counseling of NIHF in this region of Southern China. The incidence of fetal NIHF in this area is relatively high (7.9/1000). Every effort should be made to determine the underlying etiology, as it not only associates with the disease prognosis but also affects the recurrence risk. Based on our study, hematologic disease (28.4%) and chromosomal abnormalities (19.8%) are the two major causes of NIHF in this population. The mortality rate of NIHF remains unfortunately high, of which preterm birth at less than 36.5 weeks and birthweight lower than 2575 g are two poor prognostic factors for survival. The management of NIHF thus continues to be a challenge in clinics, despite of increased diagnostic rate with advanced testing technologies^25,26^.

## Methods

### Study design

In this study, we performed a retrospective review of 1004 cases with NIHF encountered in the Maternal and Children’s Hospital of Guangxi Zhuang Autonomous Region, one of the tertiary fetal medicine centers in the Southern region of China from January 2009 to June 2016. All diagnosed cases underwent detailed structural and cardiac ultrasound examinations. Trans-abdominal scans were applied by ultrasound equipment consisting of 3–5 MHz convex sector probe and GE Voluson 730 Expert (General Electric Healthcare, USA), Toshiba Aplio (Toshiba Corporation, Japan), Aloka SSD-4000 (Universal Imaging, USA) and Philips iU22 (Philips, Netherlands). HF is defined according to clinical guideline from the Society for Maternal-Fetal Medicine, and NIHF was defined as fetuses with HF but in the absence of isoimmunization.

In this work, maternal demographic features including maternal age, family history of thalassemia, parity, GA at diagnosis, NIHF etiology, delivery mode, pregnancy outcome, perinatal complications, perinatal survival, and one-year outcome were analyzed. The etiology of NIHF was grouped into 14 categories as suggested by Bellini *et al*.^1^. Our diagnostic workflow was listed as follows: 1) If both parents were found to be carriers for α or β thalassemia, genetic testing for fetal amniocentesis or cordocentesis will be offered. 2) If structural anomalies were detected and likely related to chromosomal abnormalities, fetal karyotyping and/or SNP-array would be offered and performed upon the parents’ request. 3) If other causes of hydrops than genetic factors were determined, such as congenital cystic adenomatoid malformation, the etiology was then confirmed after delivery. 4) If the cause could not be identified by scan, invasive approach for fetal karyotyping and/or SNP-array was provided. Meanwhile, the maternal blood samples were also collected and sent for hemoglobin electrophoresis examination as well as for microbiology testing to exclude parvovirus B19 and TORCH (including toxoplasmosis, rubella, cytomegalovirus, and herpes simplex). If the result of α-thalassemia or infection turns out to be positive, fetal blood sample was then collected to confirm the diagnosis. Based on these testing results, the decision of whether or not to continue the pregnancy was made by the couples after appropriate counseling.

### Statistical analysis

Data are displayed as median with interquartile range (IQR), mean ± standard deviation, or frequency (%). Kruskal-Wallis test was utilized for multiple means comparison, and proportions were compared by chi-square test. A *P*-value of < 0.05 was defined as statistically significant. All the variables remarkably associated with mortality were included in the receiver operating characteristics (ROC) curve analysis. Youden index (YI) was calculated as (specificity + sensitivity) − 1.

This work was approved by the Medical Ethics Committee of Guangxi Maternal and Children’s Hospital, and the informed consent was obtained from all participating families. All methods were performed according to the guidelines and regulations (http://www.nature.com/srep/policies/index.html#experimental-subjects).

## Supplementary information

Appendix Table 1 and Table 2
