# Supplementary material for: Etiology and Outcome of non-immune Hydrops Fetalis in Southern China: report of 1004 cases
Source: Sci Rep. 2019 Jul 24;9:10726. doi: 10.1038/s41598-019-47050-6 (PMC6656761; doi:10.1038/s41598-019-47050-6)
Supplement: Supplementary file 1 — Appendix Table 1 and Table 2 [file 41598_2019_47050_MOESM1_ESM.docx]

**Etiology and Outcome of non-immune Hydrops Fetalis in Southern China: report of 1004 cases**

**Shiyu Luo^1, 2†^, Qifei Li^3-5†^, Xuehua Hu^6†^, Lifang Wang^7^, Shuyin Tan^7^, Jiasun Su^1, 2^, Yue Zhang^1, 2^, Weijia Sun^1, 2^, Biyan Chen^1, 2^, Shen He^1, 2^, Fei Lin^1, 2^, Bobo Xie^1, 2^, Shaoke Chen^1, 2^, Pankaj B. Agrawal, MD^3-5,*^, Dahua Meng^7*^, Chunyun Fu^1, 2,6*^**

^1^Department of Genetic Metabolism, Maternal and Child Health Hospital of Guangxi Zhuang Autonomous Region, Nanning 530003, China

^2^Research Center for Guangxi Birth Defects Control and Prevention, Nanning 530003, China

^3^ Division of Genetics and Genomics, ^4^The Manton Center for Orphan Disease Research, ^5^Division of Newborn Medicine, Boston Children's Hospital and Harvard Medical School, Boston, MA 02115, USA

^6^ Medical Science Laboratory, Children’s Hospital, Maternal and Child Health Hospital of Guangxi Zhuang Autonomous Region, Nanning, China

^7^Department of Clinical Genetics, Maternal and Child Health Hospital of Guangxi Zhuang Autonomous Region, Nanning 530003, Chin

**Appendix Table 1 Detailed etiologic classifications of 1004 cases of fetal hydrops fetalis**

| **Hematologic disease (285 cases)** | | **Chromosomal abnormalities (199 cases)** | |
| --- | --- | --- | --- |
| Hemoglobin Bart’s disease (268) | --/--、αTN、βN (n = 247)  --/--、αTN、β41-42/βN (n = 7)  --/--、αTN、β17/βN (n = 5)  --/--、αTN、βE/βN (n = 3)  --/--THAI、αTN、βN (n = 3)  --/--、αTN、βIVSI-1/βN (n = 1)  --/--、αTN、β-28/βN (n = 1)  --/--、αTN、β41-42/β41-42 (n = 1) | Number (183) | 45, X (n = 83)  45, X, t(5;7) (p15;q11)mat (n = 1); 45, X mosaic (n = 3)  47, Xn, +21 (n = 49)  47, Xn, +18 (n = 29)  47, Xn, +13 (n = 11); 47, Xn, +13 mosaic (n = 1)  47, Xn, +7 mosaic (n = 1)  47, Xn, +9 mosaic (n = 1)  47, Xn, +der(22) t(11;22)(q23;q11)mat (n = 1)  47, XXX (n = 1)  92, XXYY (n = 1); 92, XXYY mosaic (n = 1) |
| Hemoglobin H diseases (17) | αα/αα、αCSα/αCSα、βN (n = 7)  αα/αα、αCSα/αCSα、β41-42/βN (n = 2)  -α3.7/--、αTN、βN (n = 2)  -α4.2/--、αTN、βN (n = 1)  -α4.2/--、αTN、β17/βN (n = 1)  αα/--、αCS、βN (n = 2)  αα/--、αQS、βN (n = 1)  αα/--、αWS、βN (n = 1) | Structure (5) | 46, Xn, +der(8)t(8;21)(q11;q11),-21 (n = 1)  46, Xn, dup(21)(q22.1q22.3) (n = 1)  46,Xn,+10, der(10;15)(q11;p11) (n = 1)  46, Xn, t(15;17)(q26;q21)dn (n = 1)  46,Xn,10q- mosaic (n = 1) |
| **Intrauterine infections (26 cases)** | Cytomegalovirus (n = 11)  Hepatitis B virus (n = 9)  Parvovirus B19 (n = 3)  Herpes simplex virus (n = 2)  Syphilis (n = 1) | CNVs (11) | arr1p36.33p36.31(791,853-7,041,175 )*1 (n = 1)  arr2q13(111398472-113100014)*1 (n = 1)  [arr5p14.3pter(38139-22612407)x1, arr13q31.2qter(89736965-115106996)x3] (n = 1)  arr 5q11.2q12.1(56368573-61428613)x1 (n = 1)  UPD (9) (n = 1)  [arr12q23.3qter(106954632-133770975)*3, arr4p15.3pter(48283-15304314)*1] (n = 1)  arr15q26.2q26.3(96057981-102397836)*1 (n = 1)  arr16q21q24.3(63863382-90130136)*2~3 (n = 1)  arr21q22.13q22.3(37872447-47526240)x3 (n = 1)  arrXp22.33(719495-1364936)*1 (n = 1)  arrYp11.21q11.23(15064733-28788643)*0 (n = 1) |

**Appendix Table 2 Top 10 ultrasound findings among 1004 NIHF cases with different etiologic classifications**

| Classifications | Ultrasound findings | Classifications | Ultrasound findings |
| --- | --- | --- | --- |
| Hematologic disease (*n* = 285) | increased cardiothoracic ratio (206), ascites (116), intestinal echo enhancement (113), pericardial effusion (113), thick placenta (107), oligohydramnios or polyhydramnios (30), skin edema (29), tricuspid regurgitation (25), cystic hygroma (24), increased MCA-PSV (23) | **Chromosomal abnormalities**  **(*n* = 199)** | skin edema (131), cystic hygroma (98), pleural effusion (39), thick nuchal transparency (38), ascites (25), pericardial effusion (24), intestinal echo enhancement (23), oligohydramnios or polyhydramnios (19), increased cardiothoracic ratio (12), thick nuchal fold (12) |
| Lymphatic anomalies (*n* = 78) | cystic hygroma (74), skin edema (21), thick nuchal transparency (10), pericardial effusion (4), thick nuchal fold (3), pyelic separation (3), lymphangioma (3), abdominal cystic mass (2), ascites (2), choroid plexus cyst (2), etc | **Cardiovascular disorders (*n* = 41)** | pericardial effusion (21), ventricular septal defect (10), increased cardiothoracic ratio (8), single umbilical artery (8), tricuspid regurgitation (8), persistent left superior vena cava (7), strong ventricular spot (7), ascites (6), pleural effusion (6), pulmonary artery stenosis (6), skin edema (6) |
| TTTS-placental/cord problems (*n* = 30) | skin edema (13), intestinal echo enhancement (8), pericardial effusion (7), oligohydramnios or polyhydramnios (7), cystic hygroma (5), increased cardiothoracic ratio (5), umbilical cord cyst (4), increased MCA-PSV (2), pleural effusion (2) | **Urinary tract malformations**  **(*n* = 29)** | pericardial effusion (16), pyelic separation (14), oligohydramnios or polyhydramnios (10), ascites (6), polycystic kidney (5), increased cardiothoracic ratio (4), intestinal echo enhancement (3), kidney agenesis (3), strong left ventricular spot (3) |
| Intrauterine infections (*n* = 26) | ascites (13), intestinal echo enhancement (8), pericardial effusion (8), pleural effusion (6), increased cardiothoracic ratio (5), thick placenta (5), oligohydramnios or polyhydramnios (4), increased MCA-PSV (3), fetal growth restriction (3), cystic hygroma (2) | **Thoracic malformations**  **(*n* = 17)** | ascites (5), intestinal echo enhancement (5), pericardial effusion (5), oligohydramnios or polyhydramnios (4), pleural effusion (4), increased cardiothoracic ratio (3), limb malformations (3), thick nuchal fold (2), thick placenta (2) |
| Gastrointestinal disorders (*n* = 7) | ascites (5), intestinal echo enhancement (5), pericardial effusion (1), pleural effusion (1), polyhydramnios (1), single umbilical artery (1), skin edema (1), strong left ventricular spot (1) | **Miscellaneous**  **(*n* = 8)** | ascites (4), pericardial effusion (4), skin edema (3), increased cardiothoracic ratio (2), intestinal echo enhancement (1), oligohydramnios (1) , polycystic kidney (1), thick nuchal fold (1), thick placenta (1), tricuspid regurgitation (1) |
| Syndromic diseases (*n* = 2) | holoprosencephaly and omphalocele (1), polyhydramnios (1), skin edema (1) | **Idiopathic causes (*n* = 282)** | ascites (89), pericardial effusion (84), intestinal echo enhancement (53), pleural effusion (52), skin edema (49), increased cardiothoracic ratio (45), oligohydramnios or polyhydramnios (44), thick placenta (34), strong left ventricular spot (20), thick nuchal fold (18), limb malformations (12) |
